# Searching for Intrinsic Causality between Colonic Dysbiosis and Non-Ischemic Cardiomyopathy: A Mendelian Randomization-Based Analysis

**DOI:** 10.3390/jcdd10100420

**Published:** 2023-10-07

**Authors:** Bin Qi, Zhi-Jie Yang, Nan Huang, Wen-Bo Zheng, Chun Gui

**Affiliations:** 1 Department of Cardiology, The First Affliated Hospital of Guangxi Medical University, Nanning 530021, China; aishizhu@126.com (B.Q.); yfyyzj@163.com (Z.-J.Y.); huangnan1202@163.com (N.H.); zwb80735816@126.com (W.-B.Z.); 2 Guangxi Key Laboratory Base of Precision Medicine in Cardiocerebrovascular Diseases Control and Prevention, Nanning 530021, China; 3 Guangxi Clinical Research Center for Cardiocerebrovascular Diseases, Nanning 530021, China

**Keywords:** mendelian randomization analysis, gut microbiota, cardiomyopathy, ischemic cardiomyopathy, non-ischemic cardiomyopathy

## Abstract

**Objective**: Little is known about gut microbiota (GM) and cardiomyopathy. Their causal relationship was explored using two-sample Mendelian randomization (TSMR) performed by R software. **Methods**: The single nucleotide polymorphisms (SNPs) were further screened based on the genome-wide association studies (GWAS) of gut microbiota and cardiomyopathy obtained from an open database. TSMR was performed using an MR-Egger regression, simple estimator based on mode, weighted median method, inverse variance weighted (IVW), weighted estimator and CML-MA-BIC to explore the causal association. And the sensitivity analysis was carried out using an MR-Egger regression and the leave-one-out sensitivity test. **Results**: As for 211 GM taxa, IVW results confirmed that the class Actinobacteria (*OR* = 0.81, *p* = 0.021) and genus Coprobacter (*OR* = 0.85, *p* = 0.033) were protective factors for cardiomyopathy. The phylum Firmicutes (*OR* = 0.87, *p* < 0.01), family Acidaminococcaceae (*OR* = 0.89, *p* < 0.01), genus Desulfovibrio (*OR* = 0.92, *p* = 0.030) and genus Prevotella9 (*OR* = 0.93, *p* = 0.029) were protective factors for ischemic cardiomyopathy. The family Rhodospirillaceae (*OR* = 1.06, *p* = 0.036) and genus Turicibacter (*OR* = 1.09, *p* = 0.019) were risk factors for ischemic cardiomyopathy. The genus Olsenella (*OR* = 0.91, *p* = 0.032) was a protective factor for non-ischemic cardiomyopathy. The order Rhodospirillales (*OR* = 1.14, *p* = 0.024), family Rikenellaceae (*OR* = 1.21, *p* = 0.012) and genus Gordonibacter (*OR* = 1.12, *p* = 0.019) were risk factors for non-ischemic cardiomyopathy. The robustness of MR results was reflected in the heterogeneity (*p* > 0.05) and pleiotropy (*p* > 0.05) analyses. **Conclusions**: A potential causal relationship of cardiomyopathy with some GM taxa has been confirmed in the current study.

## 1. Introduction

Cardiomyopathy is a collective term for a group of diseases that affect the myocardium, often leading to heart failure, and are caused by a variety of abnormal cardiac mechanical and electrical activities [1]. Cardiomyopathies are usually categorized into primary and secondary cardiomyopathies. Dilated cardiomyopathy is the main type of primary cardiomyopathy, whose primary cause is unexplained myocardial dilatation without myocardial ischemia, whereas secondary cardiomyopathy is typified by ischemic cardiomyopathy, which refers to the suppression of the blood supply to energy-dependent cardiomyocytes, and whose predominant etiological factor is coronary artery disease due to atherosclerosis [1].

ICM arises as a result of blood supply to the energy-dependent cardiomyocytes being inhibited and its most important etiology is coronary artery disease due to atherosclerosis [2]. NICM refers to cardiomyopathies that occur independently of ischemic events and are pathologically characterized by dilated cardiomyopathy with thinning of the ventricular wall (DCM) or hypertrophic cardiomyopathy with thickening of the myocardium (HCM) [3]. Although the classification of cardiomyopathy into ischemic and non-ischemic is controversial, such a classification method can be used to classify the disease by the presence of ischemia or not in order to better distinguish the etiology and plan for subsequent diagnosis and treatment [4]. These diseases are highly prevalent and predispose to recalcitrant heart failure (HF), which can cause death in patients. Many of the causes of cardiomyopathy are unknown so far [5].

Randomized controlled trials (RCTs) are the most powerful tool for proving an etiological hypothesis in epidemiological investigations. However, RCTs are limited by high requirements for study design and high costs, therefore, they are difficult to implement [6]. Confounding variables and reverse causal effects in RCTS, however, easily affect the causal link between exposure factors and disease outcome, leading to a departure in causal inference [7]. Mendelian randomization (MR), one of the more popular research methods in recent years, has the core idea that with the help of genotypes with SNPs, which will be randomly assigned to offspring individuals, it is possible to infer a causal relationship between patient exposure factors and treatment outcomes [8]. Because large sample genome-wide association studies (GWAS) are available in databases and are effective in reducing the effect of confounding factors on disease outcomes, they are widely used to determine the causal relationship between phenotype and disease [9,10].

Intestinal flora and disease, a hot topic of current research, has been shown to be closely associated with the occurrence and progression of many diseases [11,12,13,14]. Using the MR method, the relationship between gut flora and the development of cardiomyopathy was explored to find possible targets for intervention, which could help prevent the development of cardiomyopathy and improve the therapeutic efficacy.

## 2. Methods and Materials

### 2.1. GWAS Data

From a large-scale multiracial GWAS meta-analysis, the aggregated statistics for intestinal flora were collected with a total of 18,340 individuals from 211 taxa (i.e., 20 orders, 16 classes, 9 phyla, 131 genera, and 35 families) and 24 cohorts [15].

From FinnGen consortium R8 publication data, we obtained GWAS summary statistics for cardiomyopathy. This study used a “Cardiomyopathy” phenotype. The GWAS included 242,607 Finnish subjects, 5344 cases and 237,263 controls. Aggregated GWAS statistics for non-ischemic cardiomyopathy were obtained from the FinnGen consortium R8 release. In this study, a “Non-ischemic cardiomyopathy” phenotype was used. The GWAS included 285,769 Finnish subjects, 8634 cases and 277,135 controls. GWAS summary statistics for ischemic cardiomyopathy were collected from the FinnGen consortium R7 (R7 had to be used because these data were not updated in R8 and were not available). The phenotype of “ICM” was used in this study. The GWAS included 309,154 Finnish subjects, 49,030 cases and 260,124 controls. The MRC Integrative Epidemiology Unit (IEU) at the University of Bristol developed the IEU OpenGWAS project, which produced the GWAS data that were previously mentioned (https://gwas.mrcieu.ac.uk accessed on 11 July 2023). The entire GWAS summary datasets in this tool are a manually curated collection to be available for download as open-source files or through a database search of the whole data [16,17,18]. Due to potential population stratification in the dataset we selected, genomic controls were used to correct for inconsistent test statistics for each sample. The data sources for this study were based on a re-analysis on published GWAS without ethical conflict.

### 2.2. Two-Sample Mendelian Randomization (TSMR) Design

TSMR is one of several methods for MR analysis. Compared with other types of MR, TSMR can improve the efficiency of testing [19]. Additionally, published GWAS generally had a large sample size, and the number of optional instrumental variables (IVs) increased the genetic explanation for IV exposure. Exposure can be more effectively replaced by the Mendelian randomization of two samples, which is better for the precision and dependability of analytical results [20]. Additionally, genetic variation-based IVs should include the following three presumptions: the IVs we select must be conditionally independent of confounding factors, exposure (gut microbiota), and outcome (cardiomyopathy). On one hand, IVs must be strongly connected with exposure. On the other hand, IVs should not be associated with any known confounding. It can be said that IVs were independent of pleiotropy if it included the second and third hypothesis.

### 2.3. Instrument Variables (IVs)

The current work included SNPs significantly associated with the relative abundance of 211 gut microbes were selected as available IVs. Based on the three assumptions of IVs above, to ensure the close relationship between IVs and exposures (gut microbiota), IVs should first be closely tied to exposures. Given the linked characteristics of exposures (gut microbiota), second IVs should be conditionally independent of any known confounders and outcome (cardiomyopathy) [21]. The screening criteria for IVs: (1) potential instrumental variables were selected under if the significance threshold (*p* < 1.0 × 10^−5^) of single nucleotide polymorphisms (SNPs) was related to each genus within the locus range; (2) with the Thousand Genomes Project European sample data as the reference panel, the link disequilibrium (LD) among SNPs was calculated among those SNPs with *r*^2^ < 0.001 (aggregated window size = 10.000 kb), and we only retained SNPs with the lowest *p*-value; (3) single nucleotide polymorphisms with allele frequency ≤ 0.01 were removed; (4) when there were palindromic single nucleotide polymorphisms, the alleles in the forward chain were inferred by allele frequency information, and the SNPs that cannot unify the palindromic direction were deleted. In order to investigate the relationship between particular IVs and exposures, we also used a web tool to generate the F statistic [22]. An SNP with an F statistic of 10 was deleted to prevent its impact on the results if it was identified as having a weak IV bias [23].

### 2.4. MR Analysis

This study included inverse variance weighted (IVW) method [24], MR-Egger regression method [25], weighted median method [26], simple model-based estimation [27], model-based weighted estimation and CML-MA-BIC [28] were used to estimate the causal effect between immunoreaction and DCM. The most traditional approach of MR analysis uses the reciprocal of each instrumental variable’s variance as the weight to carry out weighted calculations under the assumption that all instrumental variables are effective [24]. The MR-Egger method differs from the IVW method in that the weighted regression under the condition that the instrumental variables have pleiotropy takes into consideration the existence of an intercept term. Pleiotropy degree was assessed using the intercept term, with the slope representing an estimated value of the cause–effect. The weighted median method considers the problem of large differences in estimation accuracy. Similar to the IVW method, the weight of this method generally uses the inverse weight of variance for each genetic variation. It is possible to regard simple model-based estimate as weighted median estimation with the same weight, but when the estimation accuracy of various genetic variations varies greatly, such an approach would be ineffective. In contrast to the weighted median technique, which simply demands 50% of valid weight provided by genetic variation at least, a simple modality-based estimate needs at least 50% of the genetic variation to be a valid IV. A mode-based weighted median estimate can be utilized to obtain an estimate that is consistent with the final effect when at least half of the SNPs are legitimate. CML-MA-BIC is an MR method based on constrained maximum likelihood and model average, which does not rely on the InSIDE hypothesis, and was used to control the related and unrelated pleiotropic effects. All the above methods used R software version 4.1.3, TwoSample MR package version 0.5.6.

### 2.5. Analysis Result Standard

The *p* value of IVW < 0.05, others greater than 0.05 can also be regarded as positive results, but the beta value of other methods must be consistent with the direction of IVW, otherwise it was eliminated. Positive results were divided into three categories of direct causal association, possible direct causal association and potential risk factors. The analysis results of the other five models all showed a *p* < 0.05, and it was considered as having a direct causal relation between cardiomyopathy and gut microbiota. Randomly three of the other five models showed that *p* < 0.05, and it was considered that there was a feasible direct causal relation between cardiomyopathy and gut microbiota. Those that do not meet the above two conditions were seen as potential latent correlation factors for cardiomyopathy.

### 2.6. Sensitivity Analysis

After removal of outliers, we used MR-PRESSO approach to detect outliers and re-analyzed [29]. The heterogeneity test mainly tests the differences between different IVs. A greater difference between different IVs was related to a greater heterogeneity. When there was high heterogeneity (Cochran’s Q test *p* < 0.05), the very small size of the SNPs in some disease outcomes should be excluded, or the random-effects model should be used directly to estimate MR [30]. Pleiotropy test mainly tests whether horizontal pleiotropism exists between each IV. The MR-Egger method’s intercept term is a standard way to express the constant t. The absence of horizontal pleiotropism was indicated if the intercept term was very near to zero. After removing one SNP at a time, the leave-one-out sensitivity test primarily determined the combination effect of the remaining SNPs. The MR analysis results were resilient if there was no change between the entire results after eliminating a particular SNP [31]. The TwoSample MR package in the R software environment was utilized in all of the analyses. Due to a lack of SNPs (associated with cardiomyopathy) that fitted the MR study’s hypothesis, a reverse MR analysis was not carried out.

## 3. Results

### 3.1. Selection and Validation of the IVs

A total of 2077 SNPs were identified using the LD effect and palindrome quality control steps, and unknown or repeated meaningless bacterial populations were excluded. They were associated with 196 microflorae (*p* < 1 × 10^−8^). The taxa consisted of 16 classes (178 SNPs), 9 phyla (102 SNPs), 119 genera (1231 SNPs), 32 families (352 SNPs) and 20 orders (214 SNPs). Each SNP revealed adequate validity (all F >10).

### 3.2. MR Analysis

**Figure 1:**
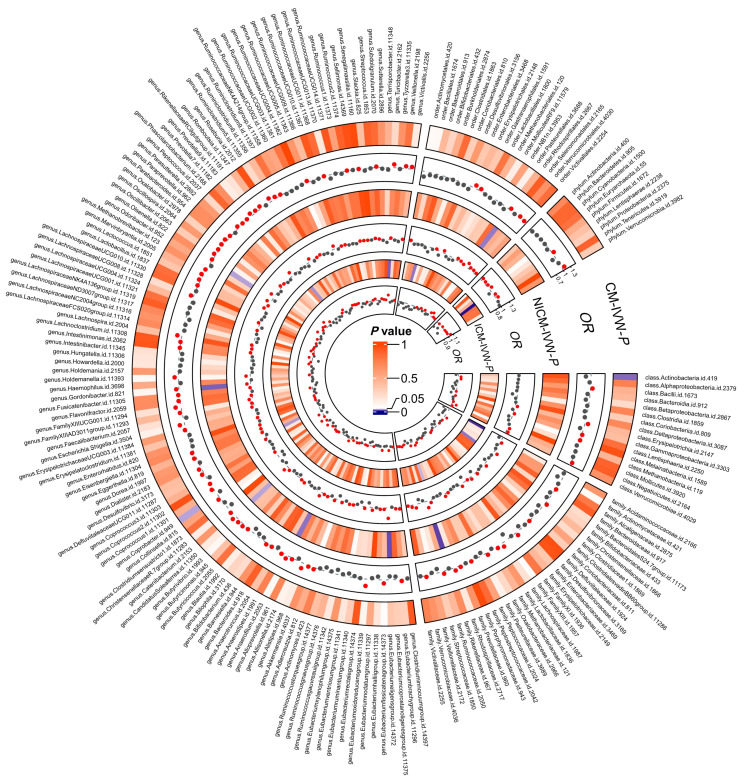
All results of MR analysis between gut microbiota and ICM, NICM and cardiomyopathy. ICM: ischemic cardiomyopathy; NICM: nonischemic cardiomyopathy; CI: confidence interval; and MR: Mendelian randomization.

**Figure 2:**
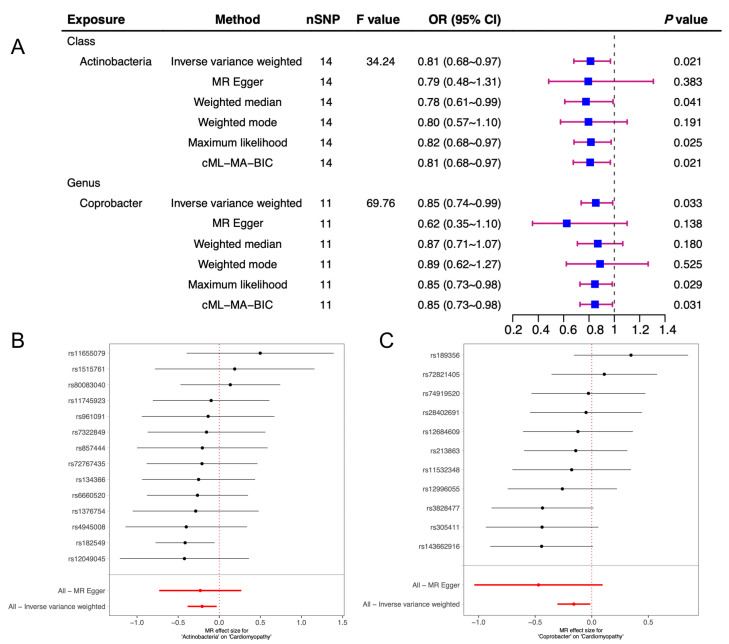
Two-sample Mendelian randomization of gut microbiota and cardiomyopathy. (**A**) After screening criteria considered significant results between gut microbiota and cardiomyopathy by MR analysis. (**B**) Forest plot of the relationship between the Actinobacteria and cardiomyopathy by MR analysis. (**C**) Forest plot of the relationship between the Coprobacter and cardiomyopathy by MR analysis. CI: confidence interval; MR: Mendelian randomization.

**Figure 3:**
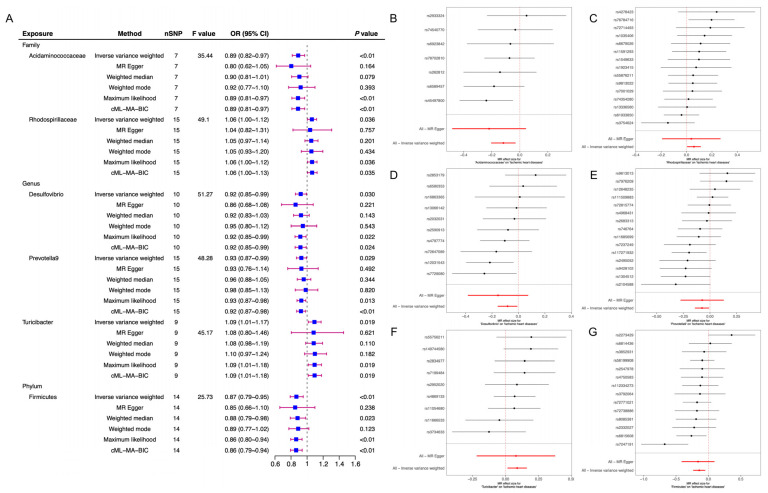
Two-sample Mendelian randomization of gut microbiota and ICM. (**A**) After screening criteria considered significant results between gut microbiota and ICM by MR analysis. (**B**) Forest plot of the relationship between the Acidaminococcaceae and ICM by MR analysis. (**C**) Forest plot of the relationship between the Rhodospirillaceae and ICM by MR analysis. (**D**) Forest plot of the relationship between the Desulfovibrio and ICM by MR analysis. (**E**) Forest plot of the relationship between the Prevotella9 and ICM by MR analysis. (**F**) Forest plot of the relationship between the Turicibacter and ICM by MR analysis. (**G**) Forest plot of the relationship between the Firmicutes and ICM by MR analysis. ICM: ischemic cardiomyopathy; CI: confidence interval; and MR: Mendelian randomization.

**Figure 4:**
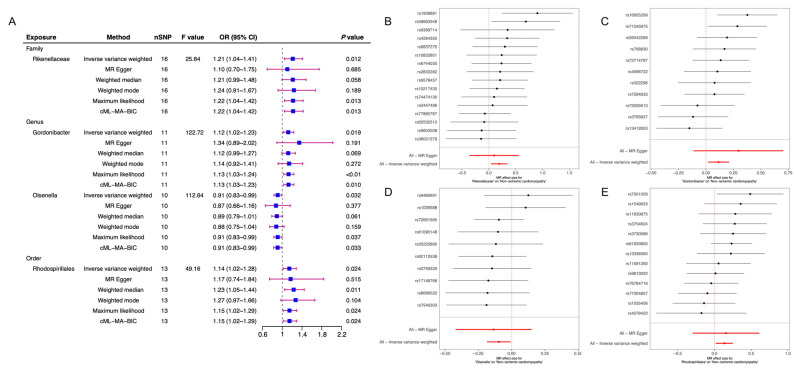
Two-sample Mendelian randomization of gut microbiota and NICM. (**A**) After screening criteria considered significant results between gut microbiota and NICM by MR analysis. (**B**) Forest plot of the relationship between the Rikenellaceae and NICM by MR analysis. (**C**) Forest plot of the relationship between the Gordonibacter and NICM by MR analysis. (**D**) Forest plot of the relationship between the Olsenella and NICM by MR analysis. (**E**) Forest plot of the relationship between the Rhodospirillales and NICM by MR analysis. NICM: nonischemic cardiomyopathy; CI: confidence interval; and MR: Mendelian randomization.

The detailed results of six TSMR analysis methods including weighted median method, IVW, simple estimator based on mode, MR-Egger regression, weighted estimator and CML-MA-BIC between 196 microflora and cardiomyopathy, ischemic cardiomyopathy and non-ischemic cardiomyopathy were shown in Figure 1 and all the details can be found in Supplementary Tables S1–S3. This study found that two intestinal microorganisms were associated with cardiomyopathy. The class Actinobacteria (*OR* = 0.81, 95%*CI*: 0.68~0.97, *p* = 0.021) and the genus Coprobacter (*OR* = 0.85, 95%*CI*: 0.74~0.99, *p* = 0.033) were both protective factors for cardiomyopathy in which the class Actinobacteria had a feasible causal link and the genus Coprobacter was a potential latent correlation factor. (Figure 2). This study also observed that six intestinal microorganisms (family Acidaminococcaceae (*OR* = 0.89, 95%*CI*: 0.82~0.97, *p* < 0.01), family Rhodospirillaceae (*OR* = 1.06, 95%*CI*: 1.00~1.12, *p* = 0.036), genus Desulfovibrio (*OR* = 0.92, 95%*CI*: 0.85~0.99, *p* = 0.030), genus Prevotella9 (*OR* = 0.93, 95%*CI*: 0.87~0.99, *p* = 0.029), genus Turicibacter (*OR* = 1.09, 95%*CI*: 1.01~1.17, *p* = 0.019), phylum Firmicutes (*OR* = 0.87, 95%*CI*: 0.79~0.95, *p* < 0.01)) were associated with ICM (Figure 3) and four intestinal microorganisms (family Rikenellaceae (*OR* = 1.21, 95%*CI*: 1.04~1.41, *p* = 0.012), genus Gordonibacter (*OR* = 1.12, 95%*CI*: 1.02~1.23, *p* = 0.019), genus Olsenella (*OR* = 0.91, 95%*CI*: 0.83~0.99, *p* = 0.032), order Rhodospirillales (*OR* = 1.14, 95%*CI*: 1.02~1.28, *p* = 0.024)) were associated with NICM (Figure 4).

For ICM, the phylum Firmicutes, family Acidaminococcaceae, genus Desulfovibrio and genus Prevotella9 were protective factors, while the family Rhodospirillaceae and genus Turicibacter were risk factors. For ICM, the phylum Firmicutes was considered to have a feasible causal relationship with it, and the remaining five intestinal microorganisms were potential latent correlation. For NICM, the genus Olsenella was a protective factors, while the order Rhodospirillales, family Rikenellaceae and genus Gordonibacter were risk factors. For NICM, the order Rhodospirillales was considered to have a feasible causal relationship with it, and the remaining three intestinal microorganisms were considered to have a potential latent correlation.

### 3.3. Sensitivity Analysis

The impact of accurate MR results on cardiomyopathy, ICM and NICM has been, respectively, confirmed. There was no horizontal pleiotropy in Actinobacteria (*p* = 0.93) and Coprobacter (*p* = 0.29) for cardiomyopathy. There was no horizontal pleiotropy in Acidaminococcaceae (*p* = 0.46), Rhodospirillaceae (*p* = 0.86), Desulfovibrio (*p* = 0.53), Prevotella9 (*p* = 0.99), Turicibacter (*p* = 0.95) and Firmicutes (*p* = 0.88) for NICM. No horizontal pleiotropy was observed in Rikenellaceae (*p* = 0.68), Gordonibacter (*p* = 0.40), Olsenella (*p* = 0.78) and Rhodospirillales (*p* = 0.92) for NICM (Table 1).

We did not detect any heterogeneity in Actinobacteria (IVW: *p* = 0.93; MR Egger: *p* = 0.90) and Coprobacter (IVW: *p* = 0.44; MR Egger: *p* = 0.46) for cardiomyopathy. There was no heterogeneity in Acidaminococcaceae (IVW: *p* = 0.68; MR Egger: *p* = 0.65), Rhodospirillaceae (IVW: *p* = 0.65; MR Egger: *p* = 0.58), Desulfovibrio (IVW: *p* = 0.40; MR Egger: *p* = 0.35), Prevotella9 (IVW: *p* = 0.14; MR Egger: *p* = 0.11), Turicibacter (IVW: *p* = 0.71; MR Egger: *p* = 0.60) and Firmicutes (IVW: *p* = 0.14; MR Egger: *p* = 0.10) for ICM. There was no heterogeneity in Actinobacteria (IVW: *p* = 0.93; MR Egger: *p* = 0.90), Ruminococcaceae_UCG_011 (IVW: *p* = 0.46; MR Egger: *p* = 0.35), Coprobacter (IVW: *p* = 0.44; MR Egger: *p* = 0.46) and Adlercreutzia (IVW: *p* = 0.81; MR Egger: *p* = 0.80) for NICM (Table 2).

**Figure 5:**
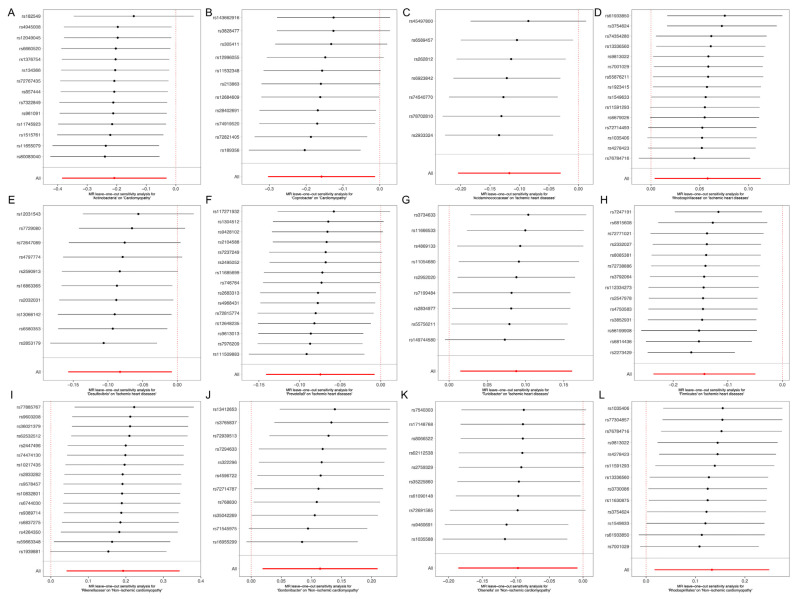
The details of sensitivity analyses for the leave-one-out approach on the association of gut microbiota on ICM, NICM and cardiomyopathy. (**A**) MR leave-one-out sensitivity analysis for ‘Actinobacteria’ on ‘Cardiomyopathy’. (**B**) MR leave-one-out sensitivity analysis for ‘Coprobacter’ on ‘Cardiomyopathy’. (**C**) MR leave-one-out sensitivity analysis for ‘Acidaminococcaceae’ on ‘lschemic heart diseases’. (**D**) MR leave-one-out sensitivity analysis for ‘Rhodospirillaceae’ on ‘lschemic heart diseases’. (**E**) MR leave-one-out sensitivity analysis for ‘Desulfovibrio’ on ‘lschemic heart diseases’. (**F**) MR leave-one-out sensitivity analysis for ‘Prevotella9’ on ‘lschemic heart diseases’. (**G**) MR leave-one-out sensitivity analysis for ‘Turicibacter’ on ‘lschemic heart diseases’. (**H**) MR leave-one-out sensitivity analysis for ‘Firmicutes’ on ‘lschemic heart diseases’. (**I**) MR leave-one-out sensitivity analysis for ‘Rikenellaceae’ on ‘Non-ischemic cardiomyopathy’. (**J**) MR leave-one-out sensitivity analysis for ‘Gordonibacter’ on ‘Non-ischemic cardiomyopathy’. (**K**) MR leave-one-out sensitivity analysis for ‘Olsenella’ on ‘Non-ischemic cardiomyopathy’. (**L**) MR leave-one-out sensitivity analysis for ‘Rhodospirillales’ on ‘Non-ischemic cardiomyopathy’. ICM: ischemic cardiomyopathy; NICM: nonischemic cardiomyopathy; CI: confidence interval; MR: Mendelian randomization.

We used MR-PRESSO to further verify that the MR results were statistically significant, so as to demonstrate the lack of horizontal pleiotropy and to guarantee the accuracy of MR-Egger regression. According to the results of MR-PRESSO (Table 3), there was no horizontal pleiotropy. Additionally, the data reliability (Figure 5) was further validated by the leave-one-out results. The IVW results were accurate when pleiotropy and heterogeneity were absent.

## 4. Discussion

Increasing gut microbes have been found to play an important role in the occurrence and development of human diseases with the in-depth development of research in the field of gut microbiota [14]. Comparing and identifying differences in gut bacteria between healthy and diseased populations is currently the focus of research on the relationship between disease and gut flora [32]. However, these different gut bacteria are not necessarily the key bacteria responsible for the development of disease. Whether changes in the gut microbiome are causal, concomitant, or completely unrelated to disease remained to be determined. Therefore, in clinical practice, the causal relationship between them must be confirmed if the changes of intestinal microbiota are to be used as an effective target for disease intervention and treatment. We used the Mendelian randomization method to find one bacterial flora showing feasible causal relationships with cardiomyopathy, ICM and NICM, and one, five, and three bacterial florae for potential latent correlation factors, respectively, on the five levels (phylum, class, order, family and genus) of 211 gut microbiota. Firmicutes showed a causal relationship related to ICM at the phylum level. Actinobacteria showed a causal relationship related to cardiomyopathy at the class level. Rhodospirillales showed a causal relationship related to NICM at the order level. No gut microbiota with feasible causal relationship to cardiomyopathy, ICM and NICM were found at the family and genus level.

The “Gut Hypothesis” is widely accepted as the link between gut microbiota and cardiomyopathy [14]. Cardiomyopathy leads to HF, which could increase intestinal venous pressure and decrease blood flow to the visceral arteries causing intestinal ischemia in patients [33]. Progressive interstitial edema and intestinal wall fibrosis are two sequelae of chronic intestinal hypoperfusion and venous congestion associated with the severity of HF in patients [12]. These structural alterations eventually result in functional alterations, such as decreased nutritional absorption and elevated gut mucosal permeability. The latter facilitates the entry of gut microbes’ byproducts into the bloodstream, such as endotoxin or lipopolysaccharide (LPS), which could cause persistent low-grade inflammation [34]. The “gut hypothesis” postulates that a “leaky” gut is a significant contributor to systemic inflammation in HF. It is suggested that the gut is the main source of systemic endotoxins since people with chronic decompensated HF have higher blood levels of LPS and anti-LPS IgA antibodies. LPS levels in the portal vein system were much greater than those in the left ventricle (LV), and these levels dropped in response to severe diuretic therapy. In addition to damaging myocardial cellular processes (such as mitochondrial function, calcium handling, etc.), causing endothelial dysfunction, lowering peripheral blood flow, and releasing systemic proinflammatory cytokines, endotoxins also directly affect cardiac function via the induction of intracardiac inflammatory responses that result in myocardial cell damage and lower cardiac contractility [35]. And changes in the intestinal flora are thought to be closely related to changes in endotoxins, which can be considered the most direct cause of heart failure due to disturbances in the intestinal flora [36].

Our research confirmed that Firmicutes were a protective factor showing a feasible causal relationship with ICM at the phylum level. Firmicutes and Bacteroides were found to be positively associated with left ventricular ejection. The risk of left ventricular hypertrophy was exacerbated by low levels of Firmicutes. Poor diastolic function was adversely correlated with high levels of Bacteroidetes. And Yang et al. also found that Bifidobacterium bifidum was closely associated with the pathogenesis of ICM in samples based on the CARDIoGRAAMplusC4D database, which has similarities with our study [37]. The left atrial diameter was correlated with a higher Firmicutes/Bacteroidetes (F/B) ratio and lower Bacteroidetes. Patients with Firmicutes and Bacteroidetes, a high F/B ratio, and Bacteroidetes had altered heart anatomy and impaired systolic and diastolic performance [38]. The specific mechanism was that the main bacteria producing butyrate in human intestinal tract were both Firmicutes and Bacteroidetes, and that Bacteroidetes mostly generated acetate and propionate. Through receptors on smooth muscle cells, butyrate and propionate can both assist in controlling vascular tone and blood pressure. By controlling the activity of intestinal macrophages and reducing the production of proinflammatory mediators brought on by lipopolysaccharide (LPS) [39], butyrate has anti-inflammatory actions and preserves the integrity of the intestinal barrier. Additionally, butyrate can promote the development of regulatory T cells, which has been demonstrated to stop the progression of HF [40]. Considering that Firmicutes had the above mechanism in the progression of HF, it was therefore considered as a protective factor in the progression of HF in cardiomyopathy, which was consistent with our findings.

Our research confirmed that Actinobacteria were a protective factor showing a feasible causal relationship to cardiomyopathy at the class level. At present, the mechanism of the protective effect of actinobacteria on cardiomyopathy was not clear. Current studies have found that actinobacteria were also a protective factor for schizophrenia, obstructive sleep apnea, Alzheimer’s disease and so on [41,42,43]. The mechanism under consideration may involve halting the inflammatory process through regulating the balance between pro- and anti-inflammatory cytokines, stabilizing the gut microbiome and intestinal permeability barrier, accelerating the immunogenicity of intestinal antigens, and altering the immunogenicity of intestinal antigens [41,44].

**Figure 6:**
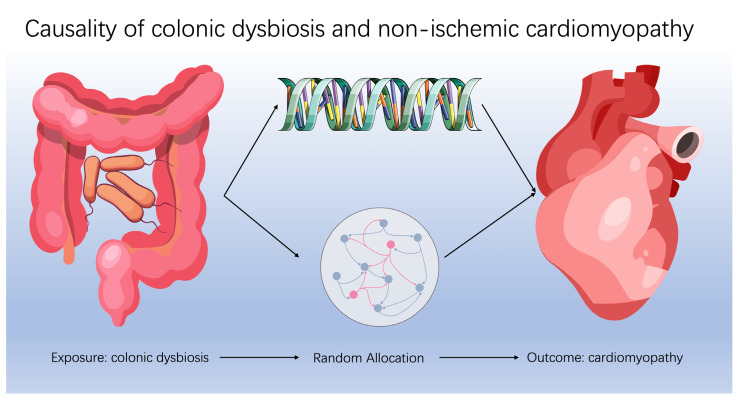
Main design and findings of the Mendelian randomization study. Intestinal flora, as an exposure factor, may contribute to non-ischemic cardiomyopathy by causing a number of factors such as genetic mutations or protein denaturation.

Our research also confirmed that Rhodospirillales were a risk factor showing a feasible causal relationship to NICM at the order level, and they were also a potential latent correlation risk factor to ICM. The relationship between Rhodospirillales and ICM and NICM has not been reported in previous studies, and its specific mechanism of action has not been fully elucidated. In addition, our study identified many more gut microbiota (Coprobacter, Acidaminococcaceae, Desulfovibrio, Prevotella9, Turicibacter, Rikenellaceae, Gordonibacter and Olsenella) as potential latent correlation factors for cardiomyopathy. Luo et al. also found a 38.1% increase in the relative risk of myocarditis and a 13.3% increase in the relative risk of hypertrophic cardiomyopathy for each one-unit increase in Shigella concentration. However, the exact mechanism remains to be explored [45]. These gut microbiotas should be further investigated to confirm the effect on cardiomyopathy and the mechanism of action (Figure 6).

There were some limitations to our study. Firstly, although we included the largest known sample size for GAWS associated with cardiomyopathy. Due to the study’s use of a European population, one drawback of GWAS may have impacted the reliability of our findings. Secondly, our findings may be affected by dietary–gene or gene–environment interactions that were difficult to entirely rule out. Thirdly, though the majority of cases and control groups were included, not all study participants, nevertheless, were exposed to rigorous quality control. Fourthly, even after Bonferroni correction, we could not discover a clear causal link between gut microbiota and cardiomyopathy, indicating that additional research was still required to establish this link. Fifthly, as in other Mendel studies, we cannot address unobserved pleiotropy. Despite these drawbacks, our study had a number of advantages. Firstly, the abundance of data in our study enabled us to examine the causal link between gut microbiota and cardiomyopathy, and the conclusions we reached were both unique and in line with previous findings. Additionally, the robustness of our findings was demonstrated by the consistent causal estimation of six methods (MR-Egger, IVW, MR-PRESO, maximum likelihood, weighted median and CML-MA-BIC).

## 5. Conclusions

Cardiomyopathy was causally associated with certain gut bacteria. But more original research was still required to determine how exactly the gut microbiota and cardiomyopathy were related. A further study should be carried out to determine the precise mechanism underlying this association.

## Figures and Tables

**Table 1:** MR results between gut microbiota and cardiomyopathy. MR, Mendelian randomization; IVW, inverse variance weighted.

ID_Exposure	ID_Outcome	Outcome	Exposure	Egger_Intercept	se	Pval
class.Actinobacteria.id.419	I9_CARDMYO	Cardiomyopathy	Actinobacteria	0.00173501	0.01841205	0.92647976
genus.Coprobacter.id.949	I9_CARDMYO	Cardiomyopathy	Coprobacter	0.03430607	0.03066712	0.29225305
family.Acidaminococcaceae.id.2166	finngen_R7_I9_ISCHHEART	Ischemic heart diseases	Acidaminococcaceae	0.01072952	0.01334403	0.45786561
family.Rhodospirillaceae.id.2717	finngen_R7_I9_ISCHHEART	Ischemic heart diseases	Rhodospirillaceae	0.00213828	0.01164505	0.85714376
genus.Desulfovibrio.id.3173	finngen_R7_I9_ISCHHEART	Ischemic heart diseases	Desulfovibrio	0.00751697	0.01143537	0.5294142
genus.Prevotella9.id.11183	finngen_R7_I9_ISCHHEART	Ischemic heart diseases	Prevotella9	−0.0001174	0.01014531	0.9909418
genus.Turicibacter.id.2162	finngen_R7_I9_ISCHHEART	Ischemic heart diseases	Turicibacter	0.00101386	0.01577514	0.95055273
phylum.Firmicutes.id.1672	finngen_R7_I9_ISCHHEART	Ischemic heart diseases	Firmicutes	0.00143736	0.00954687	0.88282534
family.Rikenellaceae.id.967	I9_NONISCHCARDMYOP	Non-ischemic cardiomyopathy	Rikenellaceae	0.00710825	0.01662915	0.67554786
genus.Gordonibacter.id.821	I9_NONISCHCARDMYOP	Non-ischemic cardiomyopathy	Gordonibacter	−0.0278034	0.03120958	0.39619398
genus.Olsenella.id.822	I9_NONISCHCARDMYOP	Non-ischemic cardiomyopathy	Olsenella	0.00555202	0.01966471	0.78485406
order.Rhodospirillales.id.2667	I9_NONISCHCARDMYOP	Non-ischemic cardiomyopathy	Rhodospirillales	−0.0023447	0.0228512	0.9201207

**Table 2:** MR results between gut microbiota and ICM and NICM. ICM: ischemic cardiomyopathy; NICM: nonischemic cardiomyopathy; MR, Mendelian randomization; and IVW, inverse variance weighted.

ID_Exposure	ID_Outcome	Outcome	Exposure	Method	Q	Q_df	Q_pval
family.Acidaminococcaceae.id.2166	finngen_R7_I9_ISCHHEART	Ischemic heart diseases	Acidaminococcaceae	MR Egger	3.356	5.000	0.645
family.Acidaminococcaceae.id.2166	finngen_R7_I9_ISCHHEART	Ischemic heart diseases	Acidaminococcaceae	IVW	4.002	6.000	0.676
family.Rhodospirillaceae.id.2717	finngen_R7_I9_ISCHHEART	Ischemic heart diseases	Rhodospirillaceae	MR Egger	11.409	13.000	0.577
family.Rhodospirillaceae.id.2717	finngen_R7_I9_ISCHHEART	Ischemic heart diseases	Rhodospirillaceae	IVW	11.442	14.000	0.651
genus.Desulfovibrio.id.3173	finngen_R7_I9_ISCHHEART	Ischemic heart diseases	Desulfovibrio	MR Egger	8.939	8.000	0.347
genus.Desulfovibrio.id.3173	finngen_R7_I9_ISCHHEART	Ischemic heart diseases	Desulfovibrio	IVW	9.422	9.000	0.399
genus.Prevotella9.id.11183	finngen_R7_I9_ISCHHEART	Ischemic heart diseases	Prevotella9	MR Egger	19.574	13.000	0.106
genus.Prevotella9.id.11183	finngen_R7_I9_ISCHHEART	Ischemic heart diseases	Prevotella9	IVW	19.574	14.000	0.144
genus.Turicibacter.id.2162	finngen_R7_I9_ISCHHEART	Ischemic heart diseases	Turicibacter	MR Egger	5.463	7.000	0.604
genus.Turicibacter.id.2162	finngen_R7_I9_ISCHHEART	Ischemic heart diseases	Turicibacter	IVW	5.467	8.000	0.707
phylum.Firmicutes.id.1672	finngen_R7_I9_ISCHHEART	Ischemic heart diseases	Firmicutes	MR Egger	18.581	12.000	0.099
phylum.Firmicutes.id.1672	finngen_R7_I9_ISCHHEART	Ischemic heart diseases	Firmicutes	IVW	18.616	13.000	0.135
family.Acidaminococcaceae.id.2166	finngen_R7_I9_ISCHHEART	Ischemic heart diseases	Acidaminococcaceae	MR Egger	3.356	5.000	0.645
family.Acidaminococcaceae.id.2166	finngen_R7_I9_ISCHHEART	Ischemic heart diseases	Acidaminococcaceae	IVW	4.002	6.000	0.676
family.Rhodospirillaceae.id.2717	finngen_R7_I9_ISCHHEART	Ischemic heart diseases	Rhodospirillaceae	MR Egger	11.409	13.000	0.577
family.Rhodospirillaceae.id.2717	finngen_R7_I9_ISCHHEART	Ischemic heart diseases	Rhodospirillaceae	IVW	11.442	14.000	0.651
genus.Desulfovibrio.id.3173	finngen_R7_I9_ISCHHEART	Ischemic heart diseases	Desulfovibrio	MR Egger	8.939	8.000	0.347
genus.Desulfovibrio.id.3173	finngen_R7_I9_ISCHHEART	Ischemic heart diseases	Desulfovibrio	IVW	9.422	9.000	0.399
genus.Prevotella9.id.11183	finngen_R7_I9_ISCHHEART	Ischemic heart diseases	Prevotella9	MR Egger	19.574	13.000	0.106
genus.Prevotella9.id.11183	finngen_R7_I9_ISCHHEART	Ischemic heart diseases	Prevotella9	IVW	19.574	14.000	0.144
genus.Turicibacter.id.2162	finngen_R7_I9_ISCHHEART	Ischemic heart diseases	Turicibacter	MR Egger	5.463	7.000	0.604
genus.Turicibacter.id.2162	finngen_R7_I9_ISCHHEART	Ischemic heart diseases	Turicibacter	IVW	5.467	8.000	0.707
phylum.Firmicutes.id.1672	finngen_R7_I9_ISCHHEART	Ischemic heart diseases	Firmicutes	MR Egger	18.581	12.000	0.099
phylum.Firmicutes.id.1672	finngen_R7_I9_ISCHHEART	Ischemic heart diseases	Firmicutes	IVW	18.616	13.000	0.135
family.Rikenellaceae.id.967	I9_NONISCHCARDMYOP	NICM	Rikenellaceae	MR Egger	11.345	14.000	0.659
family.Rikenellaceae.id.967	I9_NONISCHCARDMYOP	NICM	Rikenellaceae	IVW	11.528	15.000	0.714
genus.Gordonibacter.id.821	I9_NONISCHCARDMYOP	NICM	Gordonibacter	MR Egger	10.943	9.000	0.280
genus.Gordonibacter.id.821	I9_NONISCHCARDMYOP	NICM	Gordonibacter	IVW	11.908	10.000	0.291
genus.Olsenella.id.822	I9_NONISCHCARDMYOP	NICM	Olsenella	MR Egger	4.531	8.000	0.806
genus.Olsenella.id.822	I9_NONISCHCARDMYOP	NICM	Olsenella	IVW	4.611	9.000	0.867
order.Rhodospirillales.id.2667	I9_NONISCHCARDMYOP	NICM	Rhodospirillales	MR Egger	10.019	11.000	0.529
order.Rhodospirillales.id.2667	I9_NONISCHCARDMYOP	NICM	Rhodospirillales	IVW	10.029	12.000	0.613

**Table 3:** The outcome of MR-PRESSO.

Exposure	b	se	T-Stat	Pval
class.Actinobacteria.id.419	−0.208	0.063	−3.311	0.006
genus.Coprobacter.id.949	−0.158	0.074	−2.139	0.058
family.Acidaminococcaceae.id.2166	−0.117	0.036	−3.228	0.018
family.Rhodospirillaceae.id.2717	0.058	0.025	2.318	0.036
genus.Desulfovibrio.id.3173	−0.083	0.038	−2.165	0.059
genus.Prevotella9.id.11183	−0.074	0.034	−2.180	0.047
genus.Turicibacter.id.2162	0.088	0.031	2.847	0.022
phylum.Firmicutes.id.1672	−0.143	0.048	−3.000	0.010
family.Rikenellaceae.id.967	0.193	0.067	2.857	0.012
genus.Gordonibacter.id.821	0.114	0.049	2.347	0.041
genus.Olsenella.id.822	−0.097	0.032	−2.993	0.015
order.Rhodospirillales.id.2667	0.133	0.054	2.461	0.030

## Data Availability

The datasets generated and/or analyzed during the current study are publicly available.

## Supplementary Materials

The following supporting information can be downloaded at: https://www.mdpi.com/article/10.3390/jcdd10100420/s1. Table S1: The detailed results of six TSMR analysis methods between 196 gut microbiota and cardiomyopathy. TSMR, two-sample Mendelian randomization. Table S2: The detailed results of six TSMR analysis methods between 196 gut microbiota and ICM. TSMR, two-sample Mendelian randomization. ICM: ischemic cardiomyopathy. Table S3: The detailed results of six TSMR analysis methods between 196 gut microbiota and NICM. TSMR, two-sample Mendelian randomization. NICM: nonischemic cardiomyopathy.

## Abbreviations

GM: gut microbiota; GWAS: genome-wide association studies; DCM: dilated cardiomyopathy; ICM: ischemic cardiomyopathy; NICM: non-ischemic cardiomyopathy; MR: Mendelian randomization; TSMR: two-sample Mendelian randomization; SNP: single nucleotide polymorphism; IVW: inverse variance weighted; HF: heart failure; LPS: lipopolysaccharide; LV: left ventricle; RCTs: randomized controlled trials; LPS: lipopolysaccharide; HCM: hypertrophic cardiomyopathy; EU: integrative Epidemiology Unit; LD: link disequilibrium; and F/B: Firmicutes/Bacteroidetes.
